# Encephalopathy following ingestion of Lead-contaminated opium; magnetic resonance imaging findings

**DOI:** 10.1186/s12883-020-01750-z

**Published:** 2020-05-01

**Authors:** Maryam Haghighi-Morad, Nasim Zamani, Hossein Hassanian-Moghaddam, Maziar Shojaei

**Affiliations:** 1 Department of Radiology, Loghman-Hakim Hospital, School of Medicine, Shahid Beheshti University of Medical Sciences, Tehran, Iran; 2 Social Determinants of Health Research Center, Shahid Beheshti University of Medical Sciences, Tehran, Iran; 3 Department of Clinical Toxicology, Loghman-Hakim Hospital, School of Medicine, Shahid Beheshti University of Medical Sciences, Tehran, Iran; 4 Department of Neurology, Loghman-Hakim Hospital, School of Medicine, Shahid Beheshti University of Medical Sciences, Tehran, Iran

**Keywords:** Lead, Toxicity, Outbreak, Encephalopathy, Magnetic resonance imaging, Opioid

## Abstract

**Background:**

Encephalopathy is an uncommon but serious presentation of lead toxicity.

**Objective:**

We aimed to determine and follow-up the brain magnetic resonance imaging (MRI) abnormalities in the patients with lead encephalopathy due to ingestion of lead contaminated opium.

**Methods:**

In a cross-sectional study during lead-contaminated opium outbreak, all lead-poisoned patients with any signs/symptoms of encephalopathy were included.

**Results:**

Of 19 patients with lead encephalopathy, five died early and other five could not be sent to MRI during their hospitalization period. Mean age was 51 ± 11 years and males were dominant (89%). Median [IQR] blood lead level (BLL) was 101 [81, 108] μg/dL (range; 50 to 200 μg/dL). There was no correlation between MRI findings and signs/symptoms. MRI was normal in six and abnormal in three. Bilateral symmetric involvement of parieto-occipital lobes was observed. Gray matter, gray-white matter junction, and subcortical white matter were also affected. Follow-up MRI was performed in two with abnormal MRI which showed complete and near complete resolution of the abnormalities after cessation of opium use and treatment. *Conclusion:* There was no correlation between MRI findings and BLL. Complete recovery of brain MRI lesions was detected after cessation of opium use.

## Background

Encephalopathy is an uncommon but serious presentation of lead toxicity [1]. Lead encephalopathy is generally described by sudden commence of the manifestations including severe headache, vomiting, convulsions, mental aberration and excitement [2]. Although blood lead levels (BLLs) of higher than 100 μg/dL generally accompany with lead encephalopathy, much lower levels (as low as 38 μg/dL) may also result in encephalopathy in chronic toxicities [1]. During an outbreak in Iran, opium users were evaluated at Loghman-Hakim Hospital, and found to have high BLLs. Lead-contaminated opium was considered as the source of oral contamination [3–5]. Lead-contaminated opium has been recently discussed in the literature [6]. The objective of the current report was to determine the magnetic resonance imaging (MRI) characteristics of lead encephalopathy due to ingestion of lead-contaminated opium. We also aimed to perform a follow-up MRI after treatment in these patients to see if treatment improved their MRI abnormalities.

## Methods

### Study design and setting

This cross-sectional study was carried out during an outbreak of lead toxicity due to lead-contaminated opium in Tehran between September 2016 and August 2017 [3]. All data was pooled from a referral center for poisoned patients with annual admissions of almost 24,000 intoxicated patients supposed to be the biggest in-patient clinical toxicology center of the world [7].

### Selection of participants

All lead-poisoned patients who referred with any signs/symptoms of encephalopathy including loss of consciousness or seizure were considered as the potential participants through a chart review of the admitted patients during outbreak [3]. Diagnosis of lead toxicity was made based on BLLs higher than normal (normal range;< 10 μg/dL) checked by atomic absorption technique *(Graphite Furnace Atomic Absorption Spectrometry* [GFAAS]). After termination of treatment, BLL was followed using Lead Care II device.

Our MRI unit has recently been established with no supporting ventilator for comatose patients. Therefore, only patients who were stable and not intubated could be sent for MRI. If a comatose intubated patient improved and could be extubated, he/she would also be considered to be transferred to MRI unit where MRI was performed at the earliest convenient time. Bedside electroencephalogram (EEG) was done based on the treating physician’s decision.

Our primary outcome was MRI findings of the patients with lead encephalopathy while determination of the demographic characteristics of the patients and follow-up results of the abnormal MRIs were the secondary outcomes. MRI was performed using Vantage Elan 1.5 T MR system (Toshiba Medical Systems Corporation [TMSC]).

### Statistical analysis

For the description of quantitative variables with normal and non-normal distribution, mean (±SD) and median [IQR interquartile range] were used, respectively. For qualitative variables, percent of frequency was used. To compare normal/abnormal MRI findings with categorical variables, Fisher’s exact test was applied. For comparing continuous variables with normal/abnormal MRI findings, t-test or Mann-Whitney U test was used. A *P* value less than 0.05 was considered to be statistically significant. Statistical package for social sciences (SPSS) version 17.0 (SPSS Inc., Chicago, Ill, USA) was used for analysis.

Our institutional ethics committee approved this study.

## Results

Almost 3800 lead-poisoned opium user patients were admitted to our center [3]. Of the 19 patients who were eligible to be entered, five died early and MRI was out of service for another five patients because of technical reasons. Finally nine patients were evaluated by MRI. Lab tests for other medications/toxins that could affect brain MRI (including carbon monoxide, methanol, pesticides, cyanide, and sympathomimetics) were negative. Hypoxic encephalopathy was also excluded using cases without a period of hypoxia greater than 3 min or an out of hospital respiratory arrest as a clinical finding. Table 1 shows epidemiological characteristics of the patients.

**Table 1:** Selected characteristics of patients with lead encephalopathy

No	Age range (Year)	Gender	BLL (μg/dL)	Hgb (mg/dL)	Signs & Symptoms	EEG Interpretation	Chelating agents	MRI finding
1	50–60	M	50	12.8	Seizure, abdominal pain, agitation, constipation	mild diffuse encephalopathy	No	abnormal
2	70–80	M	200	8.7	Delirium, abdominal pain, weakness, constipation	not done	BAL + EDTA	abnormal
3	30–40	M	68	7.6	Abdominal pain, constipation, confusion, seizure	moderate diffuse encephalopathy	BAL + EDTA	abnormal
4	40–50	M	95	8.1	Abdominal pain, weakness, myalgia, confusion, seizure, insomnia, loss of appetite, dysarthria, gait disturbance, steering, delirium, seizure, constipation, ventricular tachycardia and cardiac arrest before arrival	diffuse alpha activity	BAL + EDTA	normal
5	40–50	M	101	10.2	Consciousness fluctuation, delirium, hallucination, disorientation, upper motor neuron weakness	moderate diffuse encephalopathy (cortical dysfunction)	BAL + EDTA	normal
6	50–60	M	107	8.4	Repeated seizure, weakness, agitation, loss of appetite, abdominal pain, constipation	mild diffuse cortical dysfunction	BAL + EDTA	normal
7	50–60	M	105	7.6	Abdominal pain, confusion, disorientation to time, constipation	Normal	BAL + EDTA	normal
8	40–50	M	110	10.6	Agitation, seizure, delirium	not done	BAL + EDTA	normal
9	50–60	F	> 65	9.1	Seizure, delirium, severe agitation, nausea and vomiting, confusion	mild diffuse encephalopathy	BAL + EDTA	normal

Their mean age was 51 ± 11 years (range; 39 to 72) and males were dominant (89%). Median [IQR] blood lead level was 101 [81, 108] μg/dL (range; 50 to 200) with a mean hemoglobin level of 9.2 ± 1.7 mg/dL (range; 7.6 to 12.8).

EEG was performed in three and was normal in one and abnormal in two of those who had MRIs (Table 1).

**Fig. 1:**
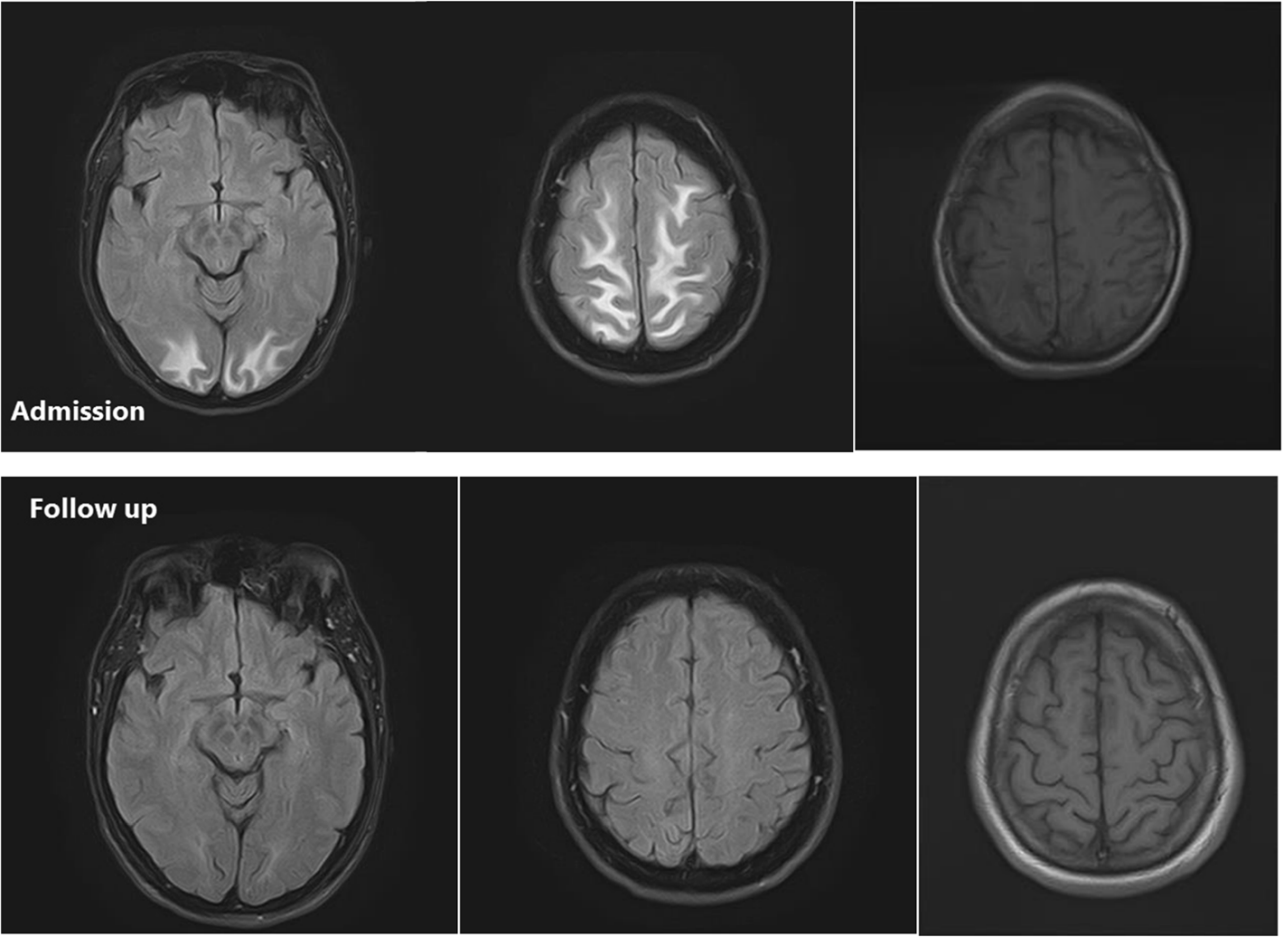
(Case 1): On admission MRI: MRI shows bilateral symmetric involvement of two areas in parasagittal parietal and occipital lobes. Gray matter, gray white matter junction, and the subcortical white matter are involved. The lesions are bright on T2-weighted and FLAIR images and hypointense on T1-weighted images. No evidence of diffusion restriction is noted. Follow-up MRI: Lesions completely resolved on the repeat MRI without chelation therapy

**Fig. 2:**
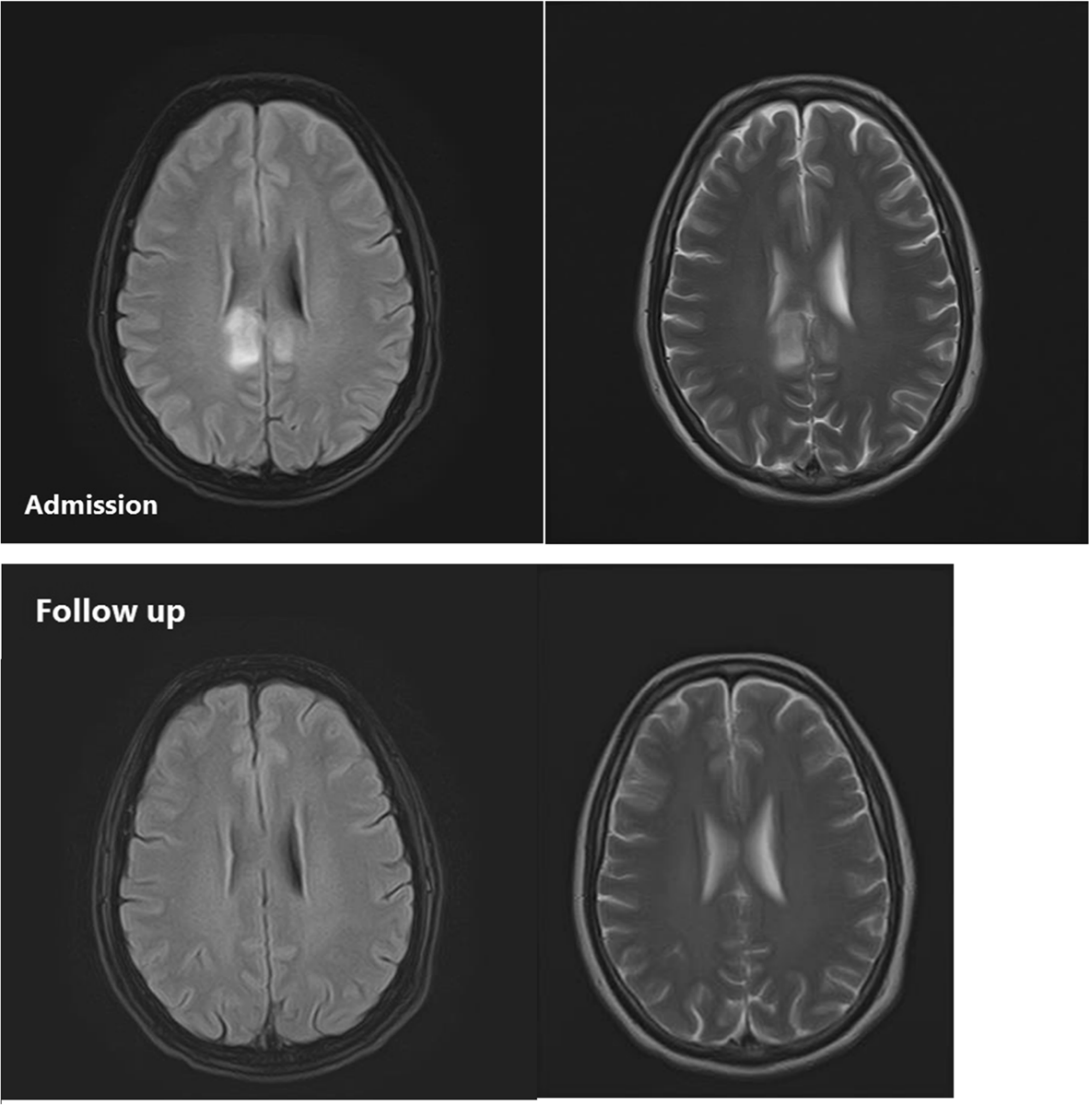
(Case 3): Admission MRI: MRI shows symmetric involvement of the parietal lobes in the parasagittal region. Gray matter, gray white matter junction, and subcortical white matter are affected. The lesions were bright on T2-weighted and FLAIR images and hypointense on T1-weighted images. Mild to moderate edema is associated with these lesions. No evidence of diffusion restriction is noted in mentioned areas. Follow-up MRI: Complete recovery of the lesions are seen in the repeat MRI after chelation therapy

**Fig. 3:**
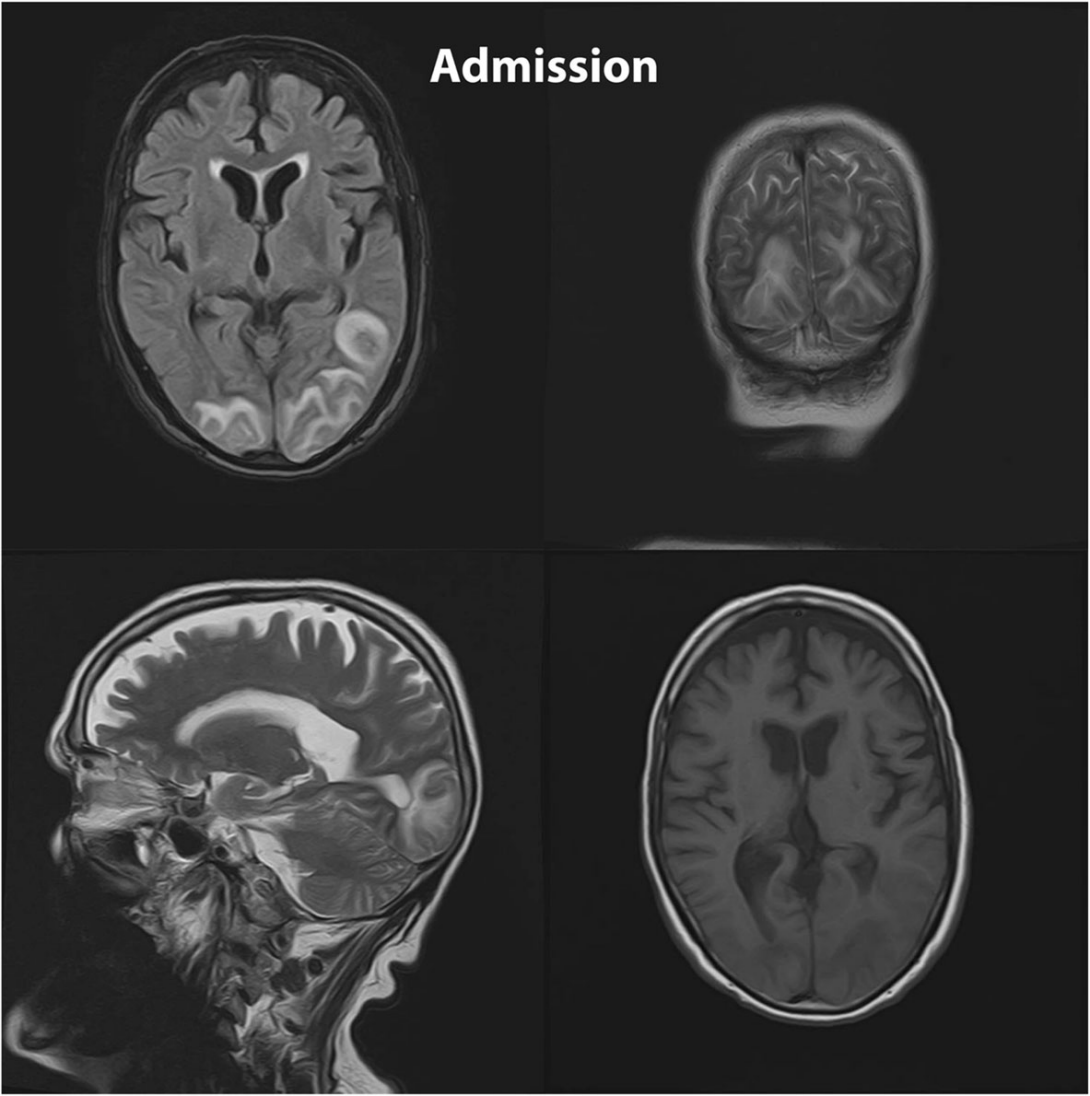
(Case 2): Admission MRI: MRI shows symmetric involvement of the parasagittal areas of bilateral parietal and occipital lobes. Asymmetric involvement of bilateral posterior temporal lobes is also evident. The affected areas are gray matter, gray white matter junction, and subcortical white matter. The lesions signal on T2-weighted and FLAIR sequences are high and low on T1-weighted sequence. No evidence of Diffusion restriction is depicted in involved areas. This patient refused follow-up MRI after chelation therapy as he believed to be completely fine

MRI was normal in six cases (67%) and showed pathologic changes in three cases (Figs. 1, 2, 3). There was no statistically significant difference between those with and without MRI abnormalities in terms of BLL, hemoglobin level, age and clinical manifestations.

Figures 1, 2, 3 show MRI findings of three patients during hospitalization. On follow-up, all patients became symptom-free after quitting the lead-contaminated opium or initiation of lead chelating agents. Two, out of three patients with abnormal MRI accepted to undergo the follow-up MRI. The time interval between the two MRIs was three and twelve months in cases 3 and 1, respectively. MRI findings resolved with no major neurological damage. BLLs were 8 and 25 μg/dL on follow-up evaluations of these two patients, respectively. We could not find any correlation between MRI findings and BLLs, age, Hb, EEG, and signs/symptoms.

## Discussion

Opioid abuse causes acute and chronic effects on neurovascular system. The most common encountered acute neurovascular effect of opioids on central nervous system is ischemia which is more often observed with intravenous injection of the drugs in comparison to oral ingestion or inhalation. The suggested mechanisms for acute ischemia due to opioids are reversible vasospasm, vasculitis and embolic events.

Reversible vasospasm is the result of stimulation of mu-opioid receptors of the vascular smooth muscles. Immune mediated response to opioids or impurities causes vasculitis. Also embolization of small insoluble particles is a potential mechanism of ischemic events due to opioids. Globus pallidus and after that hippocampus and cerebellar water shed areas are the most commonly reported location of ischemic infarctions due to opioids. Chronic neurovascular abnormalities from opioid abuse are represented with diffuse, symmetric bilateral T2W hyper intensities in the subcortical and peri ventricular white mater which are secondary to microvascular ischemic changes [8, 9]. None of these findings due to opioids toxicity are observed in our patients.

MRI findings of lead encephalopathy due to regular ingestion of opium have not been reported to date. In our study, three patients (33.3%) showed abnormal MRI findings. Atre and colleagues reported encephalopathy due to Ayurvedic medicine with bilateral symmetric involvement of the parasagittal occipital, temporal, parietal and frontal lobes and a right cerebellar lesion. In the first patient of our study, the lesions dramatically resolved without chelation therapy. This may emphasize on the importance of cessation of lead exposure as the most effective treatment in lead encephalopathy [10].

Lead encephalopathy is usually associated with BLLs higher than 100 μg/dL; but, there are few reports of encephalopathy with levels even lower than 70 μg/dL [11]. The minimum BLL in our study was 50 μg/dL and encephalopathy due to such low BLL might be due to the chronic nature of the toxicity. Lead encephalopathy can be seen even as low as 38 μg/dL in chronic cases suggesting demyelination process of lead toxicity [1]. This might be in accordance with previous study of Bouldin et al. indicating cumulative brain lead exposure and the best predictor of the prevalence of demyelination [12].

Bilateral symmetric involvement of the thalami and lentiform nuclei were reported in two patients with abnormal signal in external capsule and subcortical white matter in one patient [1]. Bilateral thalamic and T2-weighted high signal areas in the basal ganglia, posterior thalamus, pons, insula, and periventricular white matter have also been reported [11, 13]. In our patients, bilateral symmetric involvement of occipital, parietal and to a less extent, frontal and temporal lobes were observed. Gray matter, gray-white matter junction, and subcortical white matter were also affected.

Our patients’ imaging results are similar to regular findings in posterior reversible encephalopathy syndrome (PRES) which is an acute neurotoxic state characterized by temporary neurological symptoms including acute headache, altered mental status, visual loss, and coma. PRES is mainly due to hypertension but other conditions including immunosuppression therapy, hypercalcemia, and chronic renal/hepatic failure can also lead to it. It is suggested that PRES is associated with endothelial damage and blood brain barrier (BBB) disruption [14].

Interestingly, organic lead is able to cross blood-brain barrier, accumulate in the brain tissue, and mimic or mobilize calcium ions. Lead can enter the brain not only by passive transport but also as the consequence of changed BBB permeability. “Leaky microvessels” following endothelial dysfunction in lead excitotoxicity and PRES have the same MRI findings [15].

The data obtained in the study, although limited by the number of patients, is interesting because the patients’ encephalopathy improved after discontinuation of the contaminated opium. Response to treatment ruled out other opioid-related complications such as “chasing the dragon” or hypoxic encephalopathy in our patients.

### Limitations

We could only evaluate 9 out of 19 potentially eligible patients due to mortality and technical reasons. MRIs of the more severely poisoned patients would have been worse, but as these patients did not undergo MRIs, this cannot be known.

We have no access to the follow-up information of the patients that did not undergo the MRI, to know if all these patients presented reversible neurological symptoms.

## Conclusion

One-thirds of our encephalopathy patients, that could be retrieved for follow-up MRI, had reversible abnormal MRI findings. With the knowledge on our limited cases, no correlation exists between the MRI findings and BLLs or clinical signs. BBB disruption could be the same mechanism for imaging findings in lead encephalopathy and PRES.

## Data Availability

The datasets used and/or analysed during the current study are available from the corresponding author on reasonable request.

## Abbreviations

BBB: Blood brain barrier
BLL: Blood lead level
CNS: Central Nervous System
EEG: Electroencephalography
Hb: Hemoglobin
IQR: Inter Quartile Range
MRI: Magnetic Resonance Imaging
PRES: Posterior Reversible Encephalopathy Syndrome
SPSS: Statistical package for social sciences
